# Protocol for microbial profiling of low-biomass upper respiratory tract samples

**DOI:** 10.1016/j.xpro.2025.103740

**Published:** 2025-04-08

**Authors:** Mari-Lee Odendaal, Mei Ling J.N. Chu, Kayleigh Arp, Gina J. van Beveren, Giske Biesbroek, Justyna Binkowska, Thijs Bosch, James A. Groot, Raiza Hasrat, Sjoerd Kuiling, Elske M. van Logchem, Paula Lusarreta Parga, Wouter A.A. de Steenhuijsen Piters, Debby Bogaert

**Affiliations:** 1 Centre for Infectious Disease Control, National Institute for Public Health and the Environment (RIVM), Bilthoven, the Netherlands; 2 Institute for Risk Assessment Sciences (IRAS), Utrecht University, Utrecht, the Netherlands; 3 Department of Pediatric Immunology and Infectious Diseases, Wilhelmina Children’s Hospital/University Medical Center Utrecht, Utrecht, the Netherlands; 4 Spaarne Gasthuis Academy, Spaarne Gasthuis, Hoofddorp, the Netherlands; 5 Department of Pediatric Immunology, Rheumatology and Infectious Diseases, Emma Children’s Hospital, University Medical Center (Amsterdam UMC), Location Academic Medical Center (AMC), University of Amsterdam, Amsterdam, the Netherlands; 6 Centre for Inflammation Research, Institute for Regeneration and Repair, University of Edinburgh, Edinburgh, UK

**Keywords:** Bioinformatics, Sequence analysis, Health Sciences, Sequencing, Microbiology, Molecular Biology

## Abstract

The upper respiratory tract (URT) microbiota plays a role in both acute and chronic respiratory health outcomes and consists of multiple ecologically distinct niches, all of which have low bacterial biomass. Here, we present a protocol for microbial profiling of low-biomass URT samples. We describe steps for collecting, storing, and extracting DNA. We then detail procedures for performing 16S rRNA sequencing, using an Illumina MiSeq platform, to characterize microbial communities.

For complete details on the use and execution of this protocol, please refer to Odendaal et al.^1^

## Highlights

• Instructions for optimizing microbial profiling of low-biomass URT samples
• Steps to isolate DNA from URT samples using mechanical and chemical lysis
• Procedures for 16S rRNA gene V4 region sequencing and bioinformatics pipelines
• Guidance on data analysis strategies for characterizing the URT microbiota

## Abstract (editor highlights)

Publisher’s note: Undertaking any experimental protocol requires adherence to local institutional guidelines for laboratory safety and ethics.

## Graphical abstract

**Figure:**
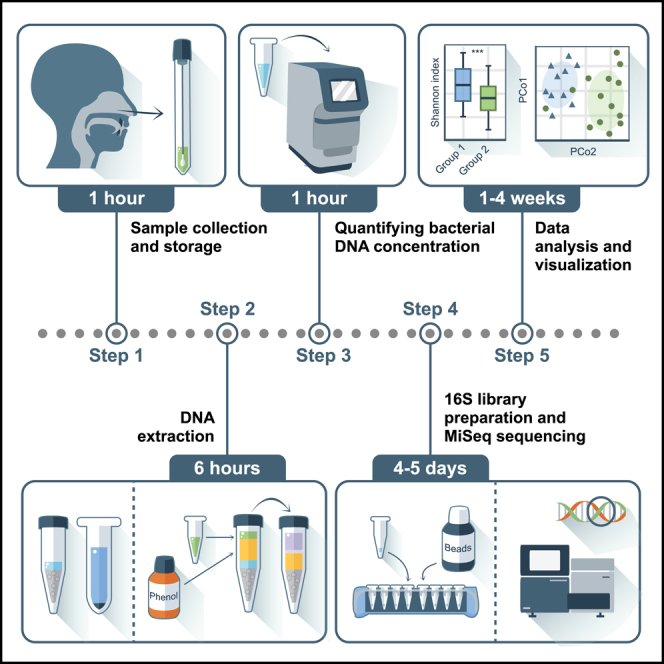

## Before you begin

The microbiome of the human URT has a considerably lower bacterial biomass compared, for example, to the well-studied gut microbiome. In particular, the nasal cavity and nasopharynx exhibit a low bacterial biomass of around 10^3^ bacteria per swab.^2^^,^^3^ Consequently, environmental contamination can become a major issue when analyzing low-biomass samples, obscuring the true microbiota signal.^4^^,^^5^ Besides, the ratio of host to bacterial DNA is also significantly higher, leading to increased contamination from host genomic DNA.^6^ To address these challenges, it is crucial to perform studies in controlled laboratory environments with standardized procedures.

This protocol was developed to investigate the microbial community composition of the human URT, focusing on saliva, oropharynx and nasopharynx samples.^7^^,^^8^^,^^9^ Unlike protocols designed for high-biomass samples like feces, which often use robots, this approach is not feasible for low-biomass samples due to excessive material loss. Laboratory procedures have first been meticulously benchmarked to accommodate low-biomass samples.^10^ Additionally, this protocol has been successfully applied to other sample types, including bronchoalveolar lavage (BAL), tracheal aspirates, nasal lavages, sputum, early life (low-biomass) feces, breast milk, vagina and skin.^7^^,^^11^^,^^12^^,^^13^^,^^14^^,^^15^ The protocol outlines detailed procedures to extract DNA from URT samples, to quantify total bacterial biomass using quantitative Polymerase Chain Reaction (qPCR), and to construct 16S rRNA gene libraries reliably. It also provides guidance on performing downstream bioinformatics and data analysis.

### Sample collection and storage

1. Collect phenotypic data where needed by for instance using questionnaires.^1^

***Note:*** Details on participants' age, sex, and the season of sampling are essential for understanding and addressing potential confounding factors, as these variables have a significant impact on microbiota composition and respiratory health outcomes.

2. Collect, preserve and store URT samples.
  a. Collect saliva samples by spitting (adults) or by placing the ORACOL (saliva collection system) between the cheek and jaw for 1 min (infants/children).
    i. Immediately squeeze the ORACOL swab to collect the saliva into a cryovial tube.
      ***Note:*** Saliva can be extracted from the ORACOL swab using one of the following methods: (1) pressing the swab against the inner wall of the provided tube and pipetting the collected saliva, or (2) inserting the sponge segment into a 10 mL syringe and extracting the saliva by pressing with the syringe plunger.
    ii. Temporarily store on dry ice or freeze immediately at −80°C.
  b. Collect nasopharynx samples by inserting a COPAN eSwab (482CE, 484CE) into the nostril, guiding it along the nasal passage to the back wall of the nasopharynx, and brush gently.
    i. Remove and submerge swabs in liquid Amies medium.
    ii. Temporarily store on dry ice or freeze immediately at −80°C.
  c. Collect oropharynx samples by gently inserting a COPAN eSwab (480CE) into the mouth and directing it to the back of the throat, avoiding contact with the oral cavity.
    i. Remove and submerge swabs in liquid Amies medium.
    ii. Temporarily store on dry ice or freeze immediately at −80°C.
      ***Note:*** Alternatively, samples can be collected using various methods and stored in different media, including DEPC-10% glycerol, or RNA protect, being mindful that this will dilute samples. A pilot study is recommended to optimize the volume of storage media. The sample collection outlined above pertains specifically to our manuscript.^11^^,^^12^
      ***Note:*** Maintaining an ultra-cold transport chain is especially critical for low-biomass samples to avoid cell death, DNA and RNA degradation, and microbiota profile shifts.
3. Transport all samples on dry ice to the laboratory and store them at −80°C.

***Note:*** It is recommended to aliquot samples before freezing. This practice helps to preserve the integrity of the sample by minimizing freeze-thaw cycles. If not applicable, aliquot with the first thaw step.

### Positive control setup

4. Prepare a 200 μL aliquot of 10^3^ diluted ZymoBIOMICS microbial community standard (whole-cell positive control) and ZymoBIOMICS microbial community DNA standard (DNA positive control).
  a. Dilute each control separately in elution buffer (QIAGEN).
5. Store the controls at −80°C until further processing.

***Note:*** Alternative positive controls containing either bacterial cells or DNA may also be used.

## Key resources table

**Table:** 

REAGENT or RESOURCE	SOURCE	IDENTIFIER
**Bacterial and virus strains**		

ZymoBIOMICS microbial community standard	Zymo Research, CA, USA	Cat#D6300
ZymoBIOMICS microbial community DNA standard	Zymo Research, CA, USA	Cat#D6306

**Biological samples**		

Copan ESwab, 482CE (transoral nasopharyngeal swab)	Copan Diagnostics Inc., CA, USA	Cat#482CE
Copan ESwab, 484CE (transnasal nasopharyngeal swab)	Copan Diagnostics Inc., CA, USA	Cat#484CE
Copan ESwab, 480CE (oropharyngeal swab)	Copan Diagnostics Inc., CA, USA	Cat#490CE
ORACOL saliva collection system	S10 Malvern Medical	

**Chemicals, peptides, and recombinant proteins**		

Actisan-5L	VWR, PA, USA	Cat#MSPP608035
Zirconium beads (0.1 mm)	Biospec Products, OK, USA	Cat#11079101z
Phenol-Tris (pH 8.0), supplemented with Tris buffer, and pre-colored with 8-hydroxyquinoline	VWR, PA, USA	Cat#108-95-2
Master mix universal TaqMan 5 × 5 mL	Thermo Fisher Scientific, MA, USA	Cat#10437304
HPLC grade water	Instruchemie, the Netherlands	Cat#2195
10 mM dNTP mix	Roche, Switzerland	Cat#11814362001
Phi-X control v.3	Illumina, CA, USA	Cat#FC-110-3001

**Critical commercial assays**		

Mini-Beadbeater-24	Biospec Products, OK, USA	Cat#112011EUR
StepOnePlus real-time PCR system	Thermo Fisher Scientific, MA, USA	Cat#4376600
Binding buffer	LGC Biosearch Technologies, Germany	Cat#NAP40102
Lysis buffer	LGC Biosearch Technologies, Germany	Cat#NAP40012
Magnetic beads solution	LGC Biosearch Technologies, Germany	Cat#NAP40137
Wash buffer 1	LGC Biosearch Technologies, Germany	Cat#NAP40181
Wash buffer 2	LGC Biosearch Technologies, Germany	Cat#NAP40211
Elution buffer	LGC Biosearch Technologies, Germany	Cat#NAP40241
LoBind Eppendorf tubes	Eppendorf	Cat#525-0130
Elution buffer	QIAGEN	Cat#19086
Phusion Hot Start II high-fidelity DNA polymerase kit (∼500 reactions)	Thermo Fisher Scientific, MA, USA	Cat#F-549L
Quant-iT PicoGreen dsDNA assay kit	Thermo Fisher Scientific, MA, USA	Cat#P7589
Kimwipes disposable wipers (lens paper)	Kimtech, USA	Cat#Z188956
AMPure XP reagent, 5 mL	Beckman Coulter, USA	Cat#A63880
MiSeq reagent kit v.3 (2 × 300 bp)	Illumina, CA, USA	Cat#MS-102-3003
Illumina MiSeq instrument	Illumina, CA, USA	Cat#SY-410-1003
DynaMag-2 magnet (magnetic separator)	Thermo Fisher Scientific, MA, USA	Cat#12321D
HLC Heating-ThermoMixer MHR 23	DITABIS, Germany	Cat#MHR 23

**Deposited data**		

Raw 16S-rRNA sequencing data	Odendaal et al.^1^; NCBI SRA	Bioproject: PRJNA997934
Silva v.138 (version 2; August 2020)	Quast et al.^16^	https://zenodo.org/record/3986799#.YfD5ti-iH0r

**Oligonucleotides**		

Forward primer 16S-F1 5′-CGA AAG CGT GGG GAG CAA A-3′	Bogaert et al.^17^	N/A
Reverse primer 16S-R1 5′-GTT CGT ACT CCC CAG GCG G-3′	Bogaert et al.^17^	N/A
Probe 16S-P1 FAM-ATT AGA TAC CCT GGT AGT CCA-ZEN	Bogaert et al.^17^	N/A
515F 16S V4 forward primer 5′-GTGCCAGC MGCCGCGGTAA-3′ (including Illumina adapters and barcodes; o6035 to o6042)	Caporaso et al.^18^	N/A
806R 16S V4 reverse primer 5′-GGACT ACHVGGGTWTCTAAT-3′ (including Illumina adapters and barcodes; o6043 to o6054)	Caporaso et al.^18^	N/A
O6056-V4-515-Read1, position 12, 100 nM TATGGTAATTGTGTGCCAGCMG CCGCGGTAA	Caporaso et al.^18^	N/A
O6057-V4-806-Read2, position 14, 100 nM AGTCAGTCAGCCGGACTA CHVGGGTWTCTAAT	Caporaso et al.^18^	N/A
O6058-V4-806-INDEX, position 13, 100 nM ATTAGAWACCCBDGTAGTCC GGCTGACTGACT	Caporaso et al.^18^	N/A

**Software and algorithms**		

R Statistical Software v.4.3.2	R Core Team	https://www.r-project.org
RStudio Server v.2023.3.0.386	RStudio	https://posit.co
Adobe Illustrator v.28.3	Adobe	https://www.adobe.com/products/illustrator.html
DADA2 v.1.26	Callahan et al.^19^	https://benjjneb.github.io/dada2/
snakemake v.5.18.1	Mölder et al.^20^	https://snakemake.readthedocs.io/en/stable/
decontam v.1.18.0	Davis et al.^21^	https://bioconductor.org/packages/release/bioc/html/decontam.html
phyloseq v.1.45.0	McMurdie & Holmes^22^	https://joey711.github.io/phyloseq/
vegan v.2.6-4	Oksanen et al.^23^	https://cran.r-project.org/web/packages/vegan/index.html
MaAsLin2 v.1.15.1	Mallick et al.^24^	https://bioconductor.org/packages/release/bioc/html/Maaslin2.html
ANCOM-BC2 v.2.4.0	Lin and Peddada^25^	https://bioconductor.org/packages/release/bioc/html/ANCOMBC.html

***Note:***Phenol is highly toxic and requires careful handling. It is recommended to always clean phenol residues with 70% ethanol v/v, as this is a recognized cleaning option. Ensure the use of special gloves that meet EN 374 standards for protection. Ensure compliance with your institute's waste management guidelines for proper handling and disposal.

## Step-by-step method details

### DNA extraction from URT samples

**Timing: 6 h**

**Figure 1:**
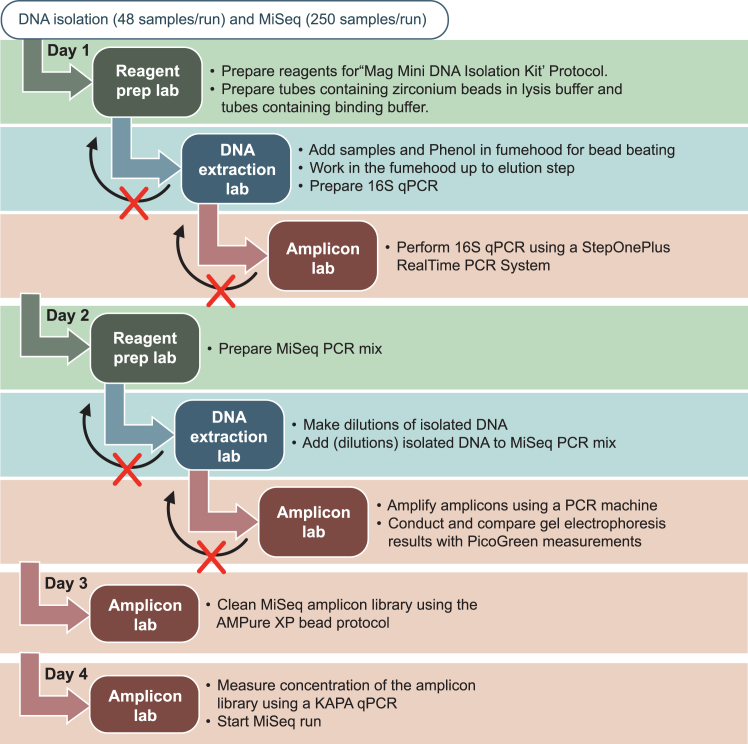
Schematic layout of work-flow through associated laboratories

**Figure 2:**
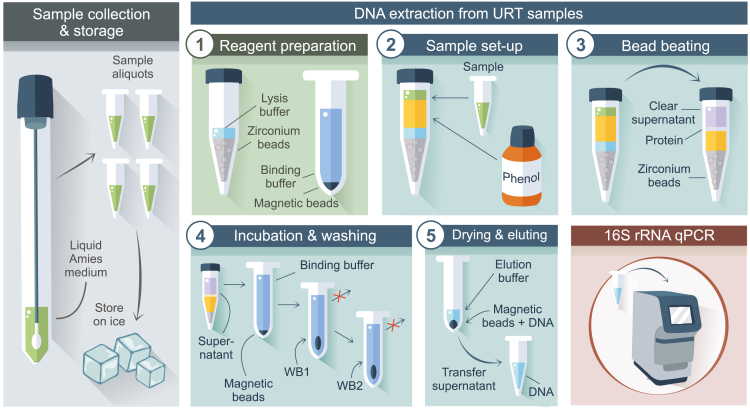
Schematic overview of the DNA extraction process

This step involves isolating DNA from URT samples using mechanical and chemical cell lysis methods. The goal is to obtain high-quality DNA for downstream analysis (Figures 1 and 2).

**CRITICAL:** To prevent contamination with unintended DNA, ensure a clean working environment by wearing gloves and thoroughly cleaning all surfaces and equipment with Actisan chloride (1 tablet per 1 L water; 0.15% Chloride w/v) / 70% ethanol v/v. Organize the workspace with only the necessary equipment. Use fresh buffers for each experiment, aliquoting them into single-use portions (one aliquot per extraction set) to prevent cross-contamination. Utilize sterile Eppendorf tubes and work in a fume hood that has been cleaned with chloride/ethanol. Always include one negative control sample per 24 samples to monitor for contamination.

***Note:*** It takes approximately 6 h to extract DNA from 48 samples. Make use of three different laboratory spaces (e.g., a reagent prep lab for reagent preparation, a DNA extraction lab for DNA extraction, and a third space for amplicon generation).

1. Reagent preparation in a dedicated **reagent prep lab.**
  **CRITICAL:** DNA, RNA, amplicons, microbial agents or patient samples are not permitted in the reagent prep lab. Only enter this lab when you have not been in laboratory spaces where microbiological or patient material was handled beforehand. It is highly recommended to schedule work in the reagent prep lab at the beginning of the day. The equipment (pipettes, vortex etc.), consumables (tubes, tips, etc.) and lab coat should not be used/worn outside of the reagent prep lab (Figure 1). Prepare single use aliquots from all your reagents: binding buffer, lysis buffer and zirconium beads, wash buffers, magnetic beads, water and elution buffer. For example, prepare aliquots with enough volume for processing 24 samples at a time. Each aliquot of reagents must be clearly labeled with the following details: reagent name, LOT/batch number, aliquoting date and expiration date. Utilize safe-lock Eppendorf tubes for smaller volume reagents and sterile Falcon tubes for larger volumes.
  a. Dispense 600 μL of 0.1 mm zirconium beads into a screw cap tube (1.5 mL) containing approximately 300 μL of lysis buffer.
    i. Add additional lysis buffer if needed (see table at step 2.d) and place the tubes in a rack.
  b. Prepare negative control tubes by adding 200 μL of lysis buffer into a 1.5 mL screw cap tube.
    ***Note:*** Per 48 samples, it is advised to include two negative controls (blanks) and one positive control containing cells (ZymoBIOMICS microbial community standard).
  c. Prepare 2 mL tubes containing magnetic bead solution in binding buffer for the second part of the extraction.
    i. Vortex the magnetic beads thoroughly.
    ii. Add 10 μL of magnetic beads followed by 1300 μL of binding buffer into a 2 mL Eppendorf tube.
      ***Note:*** Cap the tubes immediately after each magnetic bead addition and avoid contact between gloves and the inside or rim of each tube. Vortex the stock tube every 12 sample tubes to keep the beads well-mixed and evenly distributed.
2. Sample set-up in the **DNA extraction lab**.
  a. Clean the fume hood, materials and equipment (e.g., pipettes, Eppendorf racks, centrifuges, vortex) using 0.15% chloride w/v, followed by 70% ethanol v/v.
    i. Prepare separate waste bags in the fume hood (two) for phenol and (one) for biological/other waste, in accordance to your laboratory disposal guidelines.
      ***Note:*** Collect mixed phenol waste inside the working cabinet or fume hood to minimize exposure.
  b. Retrieve samples from the −80°C freezer and thaw them on ice.
  c. Label new 1.5 mL screw cap tubes with sample numbers on top. Additionally, label the 2 mL Eppendorf tubes filled with magnetic beads and binding buffer with sample numbers on both the top and side of the tubes.
    ***Note:*** These new caps will replace the old caps after step 2.d.
    ***Note:*** A minimum of four samples is necessary to balance the Beadbeater. Extensive labeling is important as the bead beating step can erase the labels.
  d. To 650 μL of 0.1 mm zirconium beads in lysis buffer (in 1.5 mL screw cap tubes):
    i. Add 550 μL of phenol (pH 8.0 with Tris) using the same pipette tip for all tubes.
      **CRITICAL:** Do not shake the bottle of phenol to avoid disturbing the buffer top layers. Pipette the phenol from the bottom layer (beneath the buffer layer).
    ii. Mix samples by gentle vortex or pipetting up and down (1000 μL pipette with filter tips) before adding it to the tubes with the zirconium beads-lysis buffer-phenol mixture. Refer to the input volumes specified for each sample type:

**Table:** 

Sample type	Input volume (μL)	Output volume (μL)
Nasopharynx	200	35–50
Oropharynx	200	50
Saliva	50 (add 150 μL extra lysis buffer at step 1.a.i)	50

3. Bead beating step.
  a. Bead beat the samples for 2 min at level 5, the highest speed (3500 strokes/min).
  b. Cool the tubes on ice for 2 min, then repeat the bead beating for another 2 min.
  c. Cool the tubes again on ice for 2 min.
    **CRITICAL:** Make sure the Beadbeater lock is well secured to avoid damage and sample spillage.
  d. Clean the Beadbeater with 70% ethanol v/v.
  e. Centrifuge the samples at 4500 × *g* for 10 min at room temperature (19°C–21°C).
    i. Put the samples in a tube rack (not on ice).
4. Incubation and washing.
  a. Transfer the upper clear supernatant (+/− 700 μL) to the 2 mL Eppendorf tubes containing magnetic beads and binding buffer.
    i. Mix thoroughly by vortexing.
      **CRITICAL:** Transfer as much of the sample as possible while avoiding the organic pellet or yellow phenol. Use a fresh pipette tip to collect any remaining sample. This step is critical for samples with low DNA concentration (<0.5 ng/μL DNA in 1 mL of sample solution).
    ii. Incubate the samples for at least 30 min in the Thermoshaker HLC set to 900 rpm at room temperature (19°C–21°C).
    iii. Disinfect the magnets of the magnetic separator rack with 70% ethanol v/v.
    iv. Spin down the tubes for 10 s at 4500 × *g*.
    v. Place the tubes in the magnet separator for 1 min to allow the beads to separate towards the magnetic separator.
    vi. Remove the supernatant and discard it in the phenol waste bag.
      ***Note:*** The isolated DNA should now be attached to the magnetic beads. While clearing the supernatant and during the wash steps, avoid disturbing the beads by keeping the pipette tip close to the opposite side of the tube.
  b. Add 300 μL of Wash Buffer 1 to the tubes.
    i. Vortex for a few seconds.
    ii. Incubate for 5 min at room temperature (19°C–21°C) in the Thermoshaker HLC.
    iii. Centrifuge the tubes for 10 s at 4500 × *g*.
    iv. Place tubes in the magnet separator for 1 min to allow the beads to attach.
    v. Remove the supernatant (pipette with a 1000 μL tip) and discard it into a non-phenol waste bag.
      ***Note:*** The supernatant should be free from phenol from this step on.
  c. Add 300 μL of Wash Buffer 2 to the tubes.
    i. Vortex for a few seconds.
    ii. Incubate for 5 min at room temperature (19°C–21°C) in the Thermoshaker HLC.
    iii. Centrifuge the tubes for 10 s at 4500 × *g*.
    iv. Place tubes in the magnet separator for 1 min to allow the beads to attach.
    v. Preheat the shaking heat block to 55°C.
    vi. Remove the supernatant (pipette with a 1000 μL tip), ensuring no buffer remains in the tubes.
      ***Note:*** Remove droplets by tapping the magnet on the workbench and using a smaller 200 μL tip to remove all leftover fluids.
5. Drying and eluting.
  a. Dry the tubes containing the magnetic beads in a Thermoshaker HLC at 55°C for 15–20 min in the fume hood, without shaking.
  b. Label new 1.5 mL Lo-Bind DNA Eppendorf tubes during the incubation.
  c. Gently tap the dry samples to ensure the magnetics beads fall to the bottom of the tube.
    ***Note:*** Dry magnetic beads will fall to the bottom when you gently tap on the tube. Do not dry the tubes longer than 25 min.
  d. Resuspend the magnetic beads in 50 μL of elution buffer (LGC) (or adjust the elution buffer amount based on experimental needs).
    **CRITICAL:** Take great care to avoid cross-contamination of the final products (eluted DNA) between samples. Utilize a printed sample list and/or arrange the tubes in separate racks to maintain organization throughout the process.
    i. Vortex the tubes for a few seconds.
    ii. Incubate the tubes for at least 15 min at 55°C while shaking at 900 rpm.
    iii. Centrifuge the tubes for 10 s at 4500 × *g*.
    iv. Place the tubes in the magnet separator for 1 min.
    v. Pipette the supernatant into the pre-labeled Lo-Bind DNA Eppendorf tubes (Step 5.b).
  e. Store the supernatant with DNA at −20°C.

### Quantifying DNA concentration using qPCR

**Timing: 1 h**

This step outlines the process for accurately measuring bacterial DNA concentration of the extracted DNA sample. This measurement is essential for ensuring the correct input amounts for downstream applications and pre-processing of the sequence data.

6. qPCR assay preparation in the **DNA extraction lab**.
  a. Prepare the qPCR assay inside a laminar airflow (LAF) cabinet accordingly:

**Table:** 

Reagents	1x mix (μL)
2x Taqman universal Master Mix	12.5
16S uni F1 (10 μM)	1
16S uni R1 (10 μM)	1
16S uni probe (5 μM)	1
H_2_O	6.5
**Subtotal**	22
Template (DNA)	3
Total reaction volume	25

**Table:** 

Name	Sequence 5′-3′	Label	Quencher	Stock (μM)	Working stock (μM)
16S uni F1	CGA AAG CGT GGG GAG CAA A	-	-	100	10
16S uni R1	GTT CGT ACT CCC CAG GCG G	-	-	100	10
16S uni probe	ATT AGA TAC CCT GGT AGT CCA	FAM	ZEN / Iowa Black FQ	50	5

7. Quantification by qPCR.
  a. Place the plate into the StepOnePlus RealTime PCR System and initiate the following program.

**Table:** 

Time	Temperature	Cycles
2 min	50°C	1 cycle
10 min	95°C	1 cycle
15 s	95°C	45 cycles
60 s	60°C

8. DNA concentration calculations.
  a. Based on a standard curve (here: Gblock Gene Fragment, starting concentration at 1 ng/μL, a 10-fold serial dilution over five times in elution buffer (LGC)), calculate the DNA concentration of all the samples and fill in worksheet “DNA isolation Run” in Table S1.
    ***Note:*** The standard curve is generated using a synthetic Gblock Gene Fragment that corresponds to the target gene being amplified with the specified primers and probe.
  b. Verify the background 16S rRNA signal in the negative extraction controls.
    i. Ensure that the actual samples have sufficient 16S rRNA yield (>0.1 pg/μL) before proceeding with further analysis. See problems 1 and 2.

### Amplicon generation for MiSeq sequencing

**Timing: 3–4 h**

This step includes amplification of the V4 region of the 16S rRNA gene using targeted primers and PCR, preparing the samples for sequencing on the Illumina Miseq platform.

9. Input DNA calculation.
  a. In Table S1, transfer the sample work number from the “DNA_isolation_Run” worksheet to the “Run_PCR_plate_X” worksheet to calculate the dilution needed to achieve a target concentration of approximately 20 pg/μL.
10. Preparation and distribution of the PCR mix and primers in the **reagent prep lab**.
  a. Thaw all reagents thoroughly.
  b. Prepare the PCR mix according:

**Table:** 

Reagents	1x MiSeq PCR mix
5x Phusion HF Buffer	5
Phusion Hot Start II High-Fidelity DNA Polymerase (F-549)	0.5
dNTPs mix (2 mM each)	2.5
H_2_O	7
Total mix volume	15

    ***Note:*** The goal is to amplify and ‘barcode’ the samples with a unique sequence of the 515F/806R primer pairs. Therefore, this mix does not include primers. Six plates with all forward and reverse primer combinations are pre-made in a concentration of 5 μM working solution.
  c. Dispense 15 μL of the MiSeq PCR mix into each well of a 96-well plate compatible with the PCR machine.
  d. Using a multichannel pipette, distribute 5 μL of the 515F-806R primer pairs into the designated wells.
  e. Add 5 μL of the mix to the designated well for the negative PCR control.
11. Proceed to the **DNA extraction lab** (in the LAF cabinet).
  a. Ensure the DNA is at the correct concentration to achieve a total template concentration of 20 pg/μL.
    i. Include a positive DNA control (ZymoBIOMICS microbial community DNA standard).
      ***Note:*** Unlike whole-cell mocks, which include intact cells to evaluate the entire workflow, the DNA mock (predefined mixture of microbial DNA) evaluate later stages, including amplification and library preparation.
  b. Add 5 μL of the (diluted) DNA (20 pg/μL) to each reaction.
  c. Run the PCR using the following program:

**Table:** 

Time	Temperature	Cycles
30 s	98°C	1 cycle
10 s	98°C	30 cycles
30 s	55°C	
30 s	72°C	
5 min	72°C	1 cycle
Infinite hold	10°C
  d. After the PCR program is complete, proceed with PicoGreen measurement and gel electrophoresis as described in the next sections.

### 16S library preparation and purification

**Timing: 2 days**

**Figure 3:**
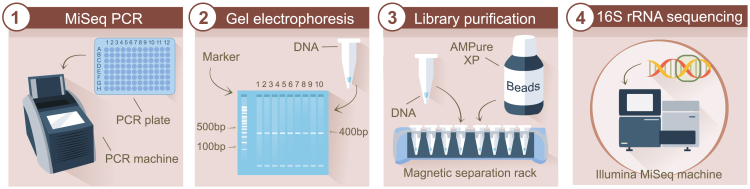
Schematic overview of the library purification and sequencing process

This step includes assessing the concentration of MiSeq PCR products with PicoGreen, followed by pooling them into a amplicon library. The pooled amplicon libraries are then purified using AMPure XP reagent (bead-based), and their concentration is measured with KAPA qPCR. These procedures are crucial in preparing the samples prior to sequencing (Figure 3).

12. Determine the concentration of MiSeq PCR products using PicoGreen.
  a. Use the setup in worksheet “Run_PCR_plate_X” in Table S1.
  b. Paste the fluorescence data from all plates into the appropriate cells, and generate the standard curve in worksheet “Plate setup & raw data” in Table S1.
  c. Perform a quality check to confirm that MiSeq PCR amplicon measurements are within the required range for downstream applications.
    i. Ensure that the R^2^ value of the standard curve is at least 0.98.
    ii. Confirm that the graph is linear and follows the equation Y = aX + b. Fill in the Y and X according to the graph in worksheet “SETUP_CALCULATION” in Table S1.
    iii. Verify that the amplicon readings fall within the range of the standard curve.
    iv. If any amplicon reading exceed the highest value on the standard curve, dilute those amplicons, re-measure, and adjust the final concentration by considering the dilution factor.
    v. Ensure that all amplicons have concentrations above 10 ng/μL, which is required for the final pool. If not, repeat the MiSeq PCR for those amplicons in a lower dilution. See worksheet “SETUP_CALCULATION” in Table S1.
      ***Note:*** If the MiSeq PCR amplicon is undiluted and contains less than 10 ng/μL, repeat the MiSeq PCR amplification to rule out errors. If sufficient material remains, repeat the DNA extraction. Otherwise, the sample will be considered a dropout. To improve DNA yield, consider performing double DNA extractions (see problem 1).
      ***Note:*** Negative PCR controls should have concentrations below 8 ng/μL. Negative DNA controls typically range between 6–15 ng/μL, but concentrations below 10 ng/μL are generally considered low. Higher concentrations as determined by the qPCR results may indicate contamination and indicate that the run should be evaluated carefully (see problem 2).
  d. Use the gel electrophoresis to verify the amplicon size, and ensure that the band size and thickness on the gel correspond to the expected amplification and PicoGreen data.
    ***Note:*** The product size of the gel electrophoresis should be approximately 400 base pairs (bp) for the V4 region of the 16S rRNA gene. Print the gel image and rename the lanes accordingly.
  e. After determining the concentration with PicoGreen, pool either 100 or 150 ng of each MiSeq PCR amplicon.
    i. Refer to the “List_view_POOL” worksheet in Table S1 for the sample volumes needed to create an equimolar pool.
      ***Note:*** The choice between 100 and 150 ng depends on the sample concentrations: for highly concentrated samples requiring small volumes, pooling at 150 ng helps minimize pipetting errors. Each pool will use a single chosen concentration. Worksheet “Submission_form” in Table S1 contains an overview of the results and measurements.
13. AMPure bead purification.
  a. Perform AMPure bead purification twice on the pooled amplicon library using a ratio of 0.9 (pool:AMPure) according to the standard protocol of the supplier.^10^
    i. Use 500 μL of the pooled amplicon library and add this to the AMPure beads.
    ii. Perform washing steps with 70% Ethanol v/v and elute in 100 μL of elution buffer (QIAGEN).
    iii. Repeat the purification steps with the 100 μL eluate as input, and finish with 35 μL of eluate.
    iv. Prepare a 10x dilution of this eluate and test it using the KAPA qPCR.

***Note:*** We perform the purification twice to ensure thorough removal of impurities and to maximize the recovery of target DNA fragments.^10^

14. Library concentration measurement with KAPA library quantification kit (Illumina platforms) and qPCR:
  a. Use the KAPA qPCR template for experimental design according to worksheet “KAPA-1 PCR setup” in Table S2.
  b. Dilute the amplicon library 100,000x and measure in triplicate.
  c. Include controls and the standard curve provided in the kit.
  d. Thaw reagents on ice and minimize their time at room temperature (19°C–21°C).
  e. Prepare the mix according to the instructions in worksheet “KAPA-1 PCR setup” in Table S2.
  f. Run the KAPA qPCR using the program specified in the same worksheet.
  g. Analyze results using worksheet “KAPA-1 analysis” in Table S2 (see problem 3).
  h. Store the library at −20°C.
  i. Dilute the library to 8 nM in elution buffer (QIAGEN) according to worksheet “load and run Miseq” in Table S2, measure again using KAPA qPCR worksheet “KAPA-2 PCR setup” and “KAPA-2 analysis”. Store at +4°C until the MiSeq run.

### MiSeq sequencing of the 16S rRNA gene

**Timing: 49–54 h**

This step ensures the generation of a high-quality 16S rRNA gene amplicon library pool and provides an overview of the Illumina MiSeq sequencing run (Figure 3).

**CRITICAL:** Before each run, check if there is enough space on the computer for the new run. One MiSeq run will produce +/− 20 GB.

***Note:*** Setting up the MiSeq takes approximately 1 h.

15. Dilute the library.
  a. Enter the concentration of the library from the second KAPA qPCR measurement in the form according to worksheet “load and run Miseq” in Table S2.
  b. Dilute the library to 4 nM in elution buffer (QIAGEN) and follow the instructions on the worksheet.
  c. Perform a maintenance wash if needed (see MiSeq Instrument).
16. Denature Phi-X control V3 to 20 pM according to the manufacturer’s protocol.
  a. Measure concentration Phi-X Control V3 using Qubit Fluorometric Quantification (Thermo Fisher scientific).
  b. In a LoBind tube, combine 10 μL of the 10 nM Phi-X Control V3 library with 15 μL of elution buffer to create a 4 nM Phi-X library.
  c. Take 5 μL of the 4 nM Phi-X solution and mix it with 5 μL of 0.2 M NaOH.
  d. Vortex the mixture, spin it down, and incubate for 5 min at room temperature (19°C–21°C).
  e. Add 5 μL of Tris solution to the mixture.
  f. Dilute by adding 985 μL of prechilled HT1 buffer to achieve a final concentration of 20 pM for the Phi-X library.
  g. Aliquot 200 μL of the solution into 5 tubes and store at −20°C.

***Note:*** The Phi-X aliquots can be stored at −20°C for (at least) up to 6 months.

17. MiSeq v3 kit cassette preparation.
  a. Remove the MiSeq v3 kit cassette from the freezer and allow it to thaw in a tray of water for at least 1.5 h.
  b. Mix the cassette by inverting it three times, and tap it to release any bubbles.
  c. Thaw the primers 06056, o6058 and o6057, which are needed for pipetting into the cassette.
  d. Prepare the cassette for primer addition. Pipette 3.4 μL of primer into the corresponding cassette position according to worksheet “load and run Miseq” in Table S2.
  e. Pipette 600 μL of the amplicon library into position 17.
18. Start the sequencing process.
  a. Select “Sequence” on the MiSeq machine and follow the program instructions.
  b. Take the flow cell and rinse it with Aqua, and dry it using lens paper, ensuring all water is removed from small openings and the flow cell is free of water stains.
  c. Carefully place the flow cell in the MiSeq machine when prompted, ensuring it is in the correct position.
  d. Load the cassette (after adding the library and primers) and the buffer bottle into their positions.
  e. Empty the waste bottle into the designated waste container.
19. Load the sample sheet with the run number, sample names and the corresponding barcodes.
20. Select the correct sample sheet in the program when prompted and continue.
21. Start the MiSeq sequencing run.
  a. Allow the MiSeq machine to verify that everything is correctly installed and available.
  b. Start the run.
    ***Note:*** A MiSeq run of 2x250 bp (V2) or 2x300 bp (V3) will take up approximately to 48–53 h.
  c. Ensure the cluster density is between 600-1000 K/mm^2^, and the Clusters Passing Filter percentage is over >85% (see problem 4).
22. Check the Phi-X percentage using Illumina MiSeq Viewer. The PhiX concentration should be between 15–25%.

***Note:*** Perform a post-wash after each sequencing run to ensure optimal cleaning of the system. Additionally, conduct a stand-by wash when no new runs are scheduled.

## Expected outcomes

This protocol is designed to enable accurate and reproducible microbial profiling of low-biomass samples from the human URT, providing insights into microbial diversity, composition, and potential associations with host factors, environmental exposures, and health outcomes. By optimizing DNA extraction, amplification, and sequencing, this protocol is expected to maximize the DNA yield and minimize contamination. Under these conditions, and following downstream quality-filtering steps described below, samples are expected to yield a sufficient number or reads per sample, enabling robust microbial analysis.

## Quantification and statistical analysis

1. After completing the MiSeq sequencing run, first perform a quality check on the files using FastQC.
2. Demultiplex the reads and generate amplicon sequence variant (ASV) count tables utilizing the DADA2 pipeline^19^ along with the latest SILVA taxonomic training set.^16^

***Note:*** ASVs offer high-resolution analogs to traditional operational taxonomic units (OTUs), providing enhanced accuracy for taxonomic classification and allowing for direct comparability across different studies.

**Figure 4:**
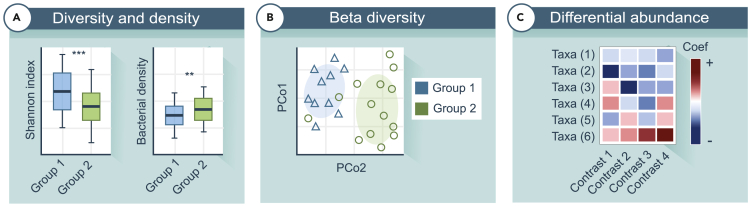
Schematic overview of microbiome analyses (A) Shannon diversity and bacterial density visualized as boxplots. Boxplots display the 25th and 75th percentiles, the median, and values within 1.5 times the interquartile range. Linear models can be used to calculate the p-values. (B) Principal coordinate analysis (PCoA). (C) Heatmap of the differential abundance analysis results. The color corresponds to the direction of the association.
  b. Use qPCR output to assess bacterial density. Normalize the data by applying a log transformation. Results can be visualized using boxplots (Figure 4A).
  c. Perform beta diversity analysis to evaluate differences in microbial community composition between samples. Use Principal Coordinates Analysis (PCA) or Non-metric Multidimensional Scaling (NMDS) based on a Bray-Curtis distance or similar indices (Figure 4B). Conduct PERMANOVA (Permutational Multivariate Analysis of Variance) to test the differences between groups and the explained variance using for instance the R package ‘vegan’.^23^
  d. Microbial clusters can be generated through complete or average linkage hierarchical clustering based on mathematic distances/dissimilarities such as Bray-Curtis, grouping participants' URT microbiota with similar microbial profiles. The optimal number of clusters can be determined using the Calinski-Harabasz and Silhouette indices. Mosaic plots can be used to visualize cluster membership and (logistic) regression analysis can be applied to statistically test the cluster membership in relation to groups of interest (e.g., age groups, health outcomes).
  e. Conduct differential abundance analyses to identify ASVs or taxa that are significantly different between experimental conditions or groups. Widely used methods include R packages ‘MaAsLin2’^24^ and ‘ANCOM-BC2’^25^ (Figure 4C).

***Note:*** QIIME/QIIME2 (Quantitative Insights Into Microbial Ecology) is a widely used and well-established bioinformatics pipeline for microbiome analysis. It is commonly implemented for tasks such as sequence quality filtering, taxonomic assignment, diversity analysis, and visualization.

3. Several pre-processing steps are necessary to remove low quality samples and contamination.
  a. Remove taxa belonging to ‘Mitochondria’, ‘Chloroplast’, ‘Archaea’, and ‘Eukaryota’ from the dataset.
  b. Rename ASVs using the lowest known taxonomic rank and indicate the rank number based on relative abundance within brackets.
  c. Eliminate taxa commonly found in negative controls, which may indicate contaminants from sources such as water, DNA extraction kits, or PCR reagents.^5^ The R package ‘decontam’ is useful for identifying contaminating sequences by analyzing their prevalence and frequency, using negative controls to help detect contaminants.^21^ Frequent found contaminants include *Comamonadaceae*, *Ralstonia*, *Thermus* and *Caulobacter.*
    ***Note:*** The ‘combined’ method of ‘decontam’ incorporates both the ‘prevalence’ and ‘frequency’ approaches. The ‘frequency’ method detects potential contaminant ASVs by looking at the inverse relation between bacterial density (qPCR) and the relative abundance of the ASVs and is recommended in combination with the ‘prevalence’ method.
  d. Generate rarefaction curves to determine the sequencing depth at which the curves plateau, indicating adequate coverage of the microbial diversity in each sample. Exclude samples with read counts below this threshold to ensure reliable downstream analyses.
4. There are various analyses that can be used to study the microbiota composition and diversity.
  a. Evaluate microbial diversity within different samples by calculating alpha diversity indices. Normalize count data by adjusting for sequencing depth. Compute various indices, such as observed species and Shannon index, using R packages like “phyloseq” and “microbiome”.^22^ Results can be visualized using boxplots for instance (Figure 4A).

## Limitations

Although this protocol offers precise, reliable, and consistent results, there are some limitations that researchers should be aware of. Despite rigorous contamination controls, low-biomass samples are inherently susceptible to contamination from reagents, laboratory environments, and handling. This susceptibility can introduce background noise that may affect the accuracy of microbial profiles, particularly for low-abundance taxa. For this reason, it is crucial to include thorough bioinformatic pre-processing steps to eliminate low-quality reads and samples. Consequently, rare or low-abundance taxa may not be consistently detectable, resulting in their potential underrepresentation. The use of 16S rRNA gene sequencing presents further challenges, as it struggles to differentiate closely related species within the same genus due to the conserved nature of the 16S rRNA gene, and it does not provide insights into microbial functional activities. To improve the detection of underrepresented or missed species (see problem 5), future research could enhance the workflow by targeting additional regions of the 16S rRNA gene, such as V5-V6 or V3-V4, or sequencing the entire 16S rRNA gene. Additionally, metagenomic approaches could offer higher taxonomic resolution and functional insights but require significantly greater sequencing depth to adequately capture the target microbiome, making them resource-intensive and challenging for niche or low-biomass samples. Overall, despite these limitations, our protocol offers a robust and highly effective approach for microbial profiling, ensuring reliable and accurate results in a wide range of applications.

## Troubleshooting

### Problem 1

The DNA yields obtained from the samples are insufficient to meet the required standards for further analysis, potentially due to challenges such as the detection of (human) mitochondrial DNA, which can dominate low-biomass samples (see step 8.b and 12.c).^6^

### Potential solution

To enhance the final DNA yield, it is recommended to perform double DNA extractions per sample. This approach is particularly relevant for ultra-low-biomass (nasal/nasopharyngeal) samples, where the initial DNA concentration is sometimes insufficient. By conducting two extractions, and combining them during wash step 1, you can maximize the extraction efficiency and ensure that sufficient DNA is obtained for subsequent analyses, thereby improving the reliability and accuracy of the results.

### Problem 2

The negative controls in the experiment have a concentration exceeding 0.1 pg/μL. This indicates (environment and/or reagent) contamination or an issue with the sample preparation, leading to unreliable results (see step 8.b and 12.c).

### Potential solution

First, review all reagents for possible contamination, ensure they are freshly prepared and stored correctly, and use high-purity or certified reagents. Next, inspect and clean instruments by thoroughly sanitizing all equipment that comes into contact with samples or reagents, and perform routine maintenance and calibration to ensure accurate measurements. Make sure all procedures are performed in a biosafety cabinet. Perform negative control runs using the same procedures and reagents to identify contaminants; analyze the results to determine if negative control concentrations exceed acceptable thresholds.

### Problem 3

The standard curve in the KAPA qPCR assay is not displaying a linear relationship (y = ax + b). In qPCR assays, a linear standard curve is essential for accurate quantification of target DNA. When the standard curve does not exhibit a linear relationship (y = ax + b), it can suggest issues with the preparation or handling of the standards. This can lead to inaccurate quantification and unreliable experimental results (see step 14.g).

### Potential solution

To address the non-linear standard curve in the KAPA qPCR assay, repeat the qPCR with careful attention to the preparation of standard dilutions. Thoroughly vortex the standard solutions to ensure uniform DNA distribution, as any concentration inconsistencies can impact linearity. After vortexing, spin down the standards to remove air bubbles and prevent inaccuracies in pipetting. Additionally, check that all pipetting steps are performed precisely. Following these steps may help achieve a more reliable standard curve and improve overall qPCR accuracy.

### Problem 4

The cluster density after sequencing is below 600 K/mm^2^ or above 1000 k/mm^2^. The cluster density of a sequencing run is critical for obtaining high-quality data. Ideally, cluster density should be maintained within the range of 600 K/mm^2^ to 1000 K/mm^2^. When the density falls below 600 K/mm^2^, there is a risk of missing bacterial clusters, leading to an underrepresentation of the sample’s diversity. Conversely, if the density exceeds 1000 K/mm^2^, the sequencing process may become imprecise, as the clusters are too dense, causing overlapping signals that can distort data accuracy (see step 21.c).

### Potential solution

Start by examining the results from positive control samples. These controls should yield expected cluster densities and sequence quality. If the results are outside the anticipated range, this may indicate an issue with the run. If the positive control results are unexpected, consider repeating the MiSeq run, starting with the KAPA quantification, as inaccuracies in the KAPA results can lead to misinterpretation of the library concentration. This, in turn, could result in overloading or underloading the MiSeq run, affecting cluster density. Note that while the final concentration selected for the library itself does not directly affect cluster density, errors in the KAPA quantification process could indirectly influence it by misguiding the dilution steps. Evaluate the loading conditions of the run; check if the flow cell was adequately loaded. Additionally, ensure the PhiX percentage is between 10–25%, as this serves as a quality control of the MiSeq run to compensate for low base diversity.

### Problem 5

Unable to detect certain taxa with the 16S rRNA gene V4 region. For instance, *Cutibacterium* frequently appears underrepresented in skin microbiome samples using this specific region for sequencing. Similarly, *Mycoplasma pneumoniae* was not detectable in the URT microbiota when using the V4 region (see limitations).

### Potential solution

Consider targeting alternative hypervariable regions of the 16S rRNA gene, such as V1-V3 or V5-V6. Combining different regions can provide a more comprehensive view of microbial diversity and abundance, as some genera may be better represented in these regions. Additionally, using a combination of 16S rRNA sequencing with other molecular techniques (e.g., qPCR for specific taxa) can enhance the detection of underrepresented taxa.

## Resource availability

### Lead contact

Further information and requests for resources and reagents should be directed to and will be fulfilled by the lead contact, Debby Bogaert (D.Bogaert@ed.ac.uk).

### Technical contact

Technical questions on executing this protocol should be directed to and will be answered by the technical contact, Mei Ling Chu (mei.ling.chu@rivm.nl).

### Materials availability

This study did not generate any unique materials or reagents.

### Data and code availability

The paired FASTQ files containing 16S-rRNA sequencing data have been submitted to the NCBI Sequence Read Archive (SRA) under accession number Bioproject: PRJNA997934. The code used for data processing and microbiome analysis is available on GitLab: https://gitlab.com/Mari-Lee/URT_microbiota_baseline.

## Acknowledgments

The work described in the lead article^1^ was conducted as part of the National Institute for Public Health and the Environment (RIVM) strategic project TRIuMPH. The serosurveys in the Netherlands (PIENTER-3) and in the Caribbean Netherlands (HSCN) were led by RIVM in close partnership with local Public Health Services (GGD) and Statistics Netherlands (CBS). We extend our gratitude to all volunteers who participated in this study. This work was partially supported by the Netherlands Organization for Scientific Research (NWO-VIDI; grant number 91715359, awarded to D.B.) and the Chief Scientist Office/NHS Research Scotland through a Scottish Senior Clinical Fellowship award (SCAF/16/03, awarded to D.B.).

## Author contributions

M-L.O. and M.L.J.N.C. were responsible for the writing of the original draft. M.L.J.N.C., K.A., G.B., J.A.G., R.H., S.K., E.M.v.L., and P.L.P. were responsible for the optimization, execution, and quality control of the laboratory work. M.-L.O., G.J.v.B., J.B., and W.A.A.d.S.P. supported the development and execution of the bioinformatics workflows. D.B. was central to conceptualizing the protocol, guiding its development and execution, and securing the majority of the funding. T.B. contributed to the protocol validation and supported funding acquisition. All authors critically revised the manuscript for important intellectual content and approved the final manuscript.

## Declaration of interests

The authors declare no competing interests.

## Supplemental information

Table S1. Setup for DNA extraction until MiSeq PCR, related to steps 8–12

Table S2. Setup for KAPA qPCR and MiSeq run, related to steps 14–17
